# The initiator methionine tRNA drives cell migration and invasion leading to increased metastatic potential in melanoma

**DOI:** 10.1242/bio.019075

**Published:** 2016-08-19

**Authors:** Joanna Birch, Cassie J. Clarke, Andrew D. Campbell, Kirsteen Campbell, Louise Mitchell, Dritan Liko, Gabriela Kalna, Douglas Strathdee, Owen J. Sansom, Matthew Neilson, Karen Blyth, Jim C. Norman

**Affiliations:** Beatson Institute for Cancer Research, Garscube Estate, Glasgow G61 1BD, Scotland

**Keywords:** Cell migration, Invasion, tRNA repertoire, RNA polymerase III (Pol III), tRNA_i_^Met^, Integrin, Extracellular matrix, Melanoma, Metastasis

## Abstract

The cell's repertoire of transfer RNAs (tRNAs) has been linked to cancer. Recently, the level of the initiator methionine tRNA (tRNA_i_^Met^) in stromal fibroblasts has been shown to influence extracellular matrix (ECM) secretion to drive tumour growth and angiogenesis. Here we show that increased tRNA_i_^Met^ within cancer cells does not influence tumour growth, but drives cell migration and invasion via a mechanism that is independent from ECM synthesis and dependent on α5β1 integrin and levels of the translation initiation ternary complex. *In vivo* and *ex vivo* migration (but not proliferation) of melanoblasts is significantly enhanced in transgenic mice which express additional copies of the tRNA_i_^Met^ gene. We show that increased tRNA_i_^Met^ in melanoma drives migratory, invasive behaviour and metastatic potential without affecting cell proliferation and primary tumour growth, and that expression of RNA polymerase III-associated genes (which drive tRNA expression) are elevated in metastases by comparison with primary tumours. Thus, specific alterations to the cancer cell tRNA repertoire drive a migration/invasion programme that may lead to metastasis.

## Abstract (precis)

**Summary:** This paper demonstrates a functional link between a cancer-associated alteration to the tRNA repertoire and the invasive behaviour of cancer cells by demonstrating that increased tRNA_i_^met^ can drive invasion and metastasis in melanoma cells.

## INTRODUCTION

Many of the steps to metastasis require cancer cells to migrate invasively through the extracellular matrix (ECM) that surrounds tumours and tissues (Sahai, 2005). This type of invasive migration involves coordination of a number of cellular processes, such as secretion of degradative factors (for example, matrix metalloproteases) to clear a path through the surrounding tissue, engagement of cell adhesion receptors (such as α5β1 integrin) with the ECM, and recruitment of the contractile cytoskeleton to integrins which provide traction force to translocate the invading cell away from the primary tumour. Cancer cells must invoke transcriptional programmes to coordinate these various cellular processes. Thus, in addition to upregulation of proteins that directly contribute to cell migration, the pro-invasive programme involves production of other regulators (such as transcription factors) which coordinate expression of pro-migratory and pro-invasive factors (Ozanne et al., 2000).

Pro-invasive programmes may also be implemented at the level of the ribosome. Indeed, a number of ‘ribosomopathies’ (syndromes with well-characterised inheritable mutations in ribosomal subunits or other components of the translation machinery) display a marked cancer predisposition (Ganapathi and Shimamura, 2008; Ruggero, 2013). Furthermore, key oncogenic pathways, such as the PI3K-Akt-mTOR axis, control particular components of the translational machinery, such as the eIF4E initiation factor and the S6 ribosomal protein, to modulate selectivity of mRNA translation in a way that influences cancer-relevant processes (Ruggero and Sonenberg, 2005). For example, upregulation of mTOR signalling favours translation of a number of transcripts for pro-migratory proteins (such as YB1, vimentin and CD44) and it is thought that these can cooperate to promote metastasis (Hsieh et al., 2012). A key regulator of the ribosome and the mRNA translation machinery is RNA polymerase III (Pol III). Pol III is the largest of the three eukaryotic polymerases and is responsible for transcription of a number of short, non-coding RNAs which include transfer RNAs (tRNAs) and RNA ribosomal components. Pol III function is controlled by multiple oncogenes and tumour suppressors including c-myc, p53 and Rb, mooting this RNA polymerase as a potential master regulator of a translational programme that contributes to cancer progression (White, 2008). It has been known for some time that expression of certain Pol III target genes, for instance tRNA_i_^Met^ (the initiator methionine tRNA responsible for recognising the start codon and initiating translation), is upregulated in cancer and it has been proposed that this may drive progression of the disease (Pavon-Eternod et al., 2009, 2013). More recently it has become clear that the cell's tRNA repertoire differs profoundly between different tissues (Dittmar et al., 2006) and between differentiated cells and cancer lines (Gingold et al., 2014). Indeed, proliferating cells have increased levels of tRNAs with codons that are particularly abundant in transcripts encoding proteins associated with cell growth and proliferation; thus, the tRNAome compliments transcriptional programmes to ensure efficient translation of the cohorts of transcripts which are associated with particular cellular outcomes. Consistent with previous reports, Gingold et al. report that highly proliferative cancer cell lines commonly display increased levels of tRNA_i_^Met^ (Gingold et al., 2014). Thus it would seem natural to propose that this alteration to the tRNA repertoire would drive increased use of the AUG codon leading to upregulation of translation initiation and protein synthesis to support cancer cell growth and proliferation. However, a recent study in stromal fibroblasts indicate that elevated levels of tRNA_i_^Met^ do not directly support increased cellular protein synthesis and proliferation (Clarke et al., 2016). Rather, increased tRNA_i_^Met^ alters the fibroblast secretome to favour synthesis and secretion of certain collagens (particularly type II collagen) which are incorporated into the tumour extracellular matrix (ECM). This collagen II-rich ECM supports motility of endothelial cells which leads to increased angiogenesis and tumour growth. These findings are consistent with studies in *Drosophila* indicating that increased levels of tRNA_i_^Met^ drive cellular growth via a non-cell autonomous mechanism involving alterations to the cellular secretome (Rideout et al., 2012). Indeed, introduction of an extra copy of the tRNA_i_^Met^ gene into flies is sufficient to markedly enhance larval growth which is driven by increased release of insulin-like peptides from brain neurosecretory cells which, in turn, circulate throughout the embryo to promote growth of developing organs and tissues.

Because Pol III products are thought to be increased in tumour cells (White, 2005) as well as in stromal fibroblasts (Clarke et al., 2016), we have generated approaches to manipulate levels of Pol III and its product tRNA_i_^Met^ in tumour cells and have determined the consequences of this on tumour cell behaviour. Using both *in vitro* and *in vivo* approaches we show that increased levels of tRNA_i_^Met^ in tumour cells drives metastasis by enlisting integrin-dependent cell migration and invasion without influencing cell growth or proliferation, indicating that the cell migratory machinery responds to alterations in the tRNA repertoire during the metastatic process.

## RESULTS

### tRNA_i_^Met^ drives cell migration dependent on α5β1 integrin and the translation initiation ternary complex

**Fig. 1.**
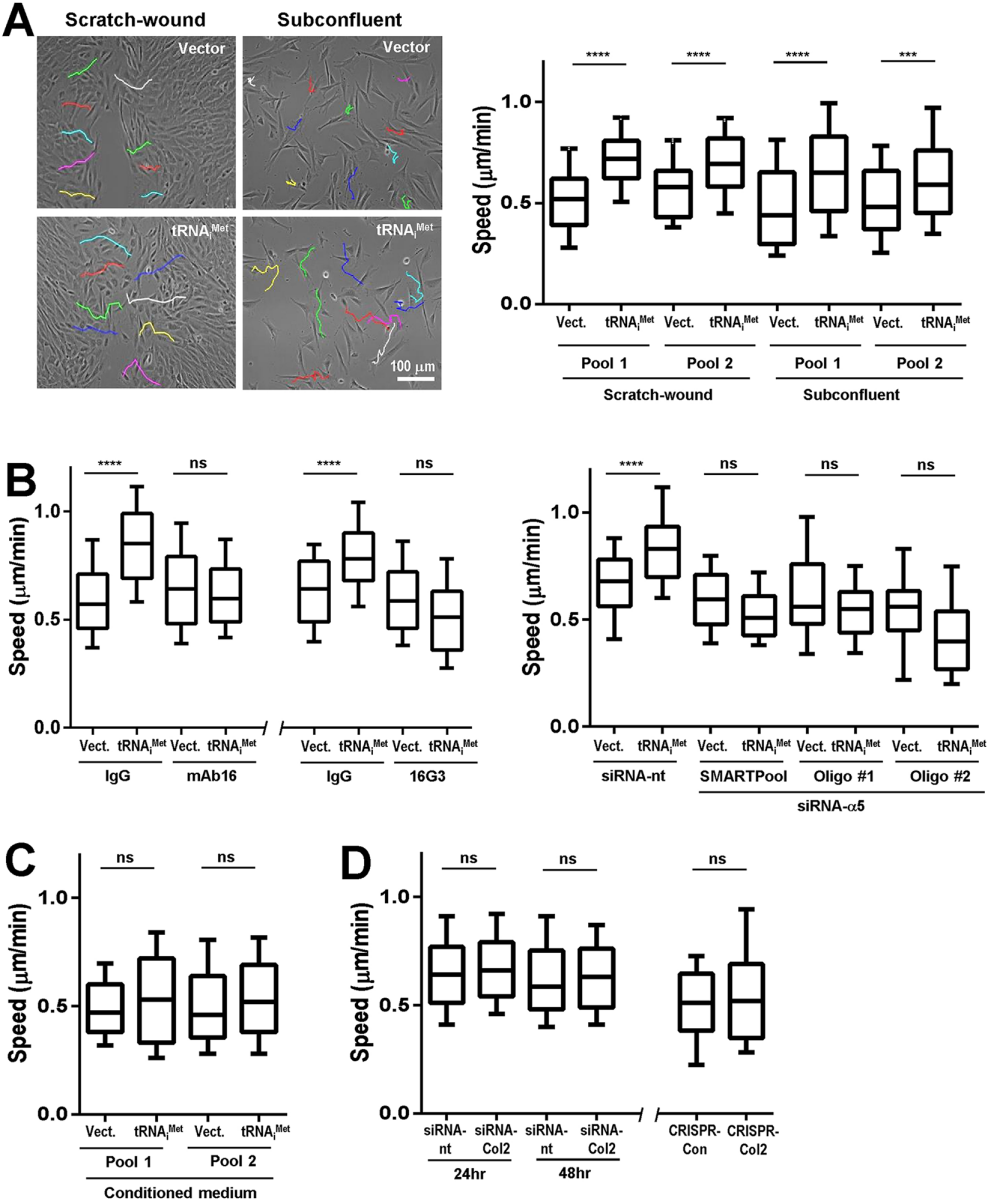
**Elevated levels of tRNA_i_^Met^ promote α5β1-dependent cell migration.** (A) Immortalised mouse embryonic fibroblasts (iMEFs) were stably transfected with a vector encoding tRNA_i_^Met^ or an empty vector control (Vect.) (2 independent pools of each). The migration speed of cells plated subconfluently, or of those moving into scratch-wounds was determined using time-lapse microscopy followed by cell tracking. Representative cell trajectories are displayed in the left panels. Data are represented as box and whisker plots (whiskers: 10-90 percentile), *n*=3 independent experiments; ****P<0.0001; ***P<0.001; Mann–Whitney test. Scale bar: 100 μm. (B) iMEFs expressing tRNA_i_^Met^ or an empty vector control (Vect.) were transfected with siRNAs targeting α5 integrin (siRNA-α5; either a SMARTPool or two individual siRNA oligonucleotides as indicated), a non-targeting control (siRNA-nt) (right panel) or were left untransfected (left and centre panels). Cell were plated onto plastic surfaces in the presence of blocking antibodies against α5 integrin (mAb16; left panel), the RGD-containing integrin-binding site in fibronectin (16G3; centre panel) or the appropriate isotype-matched control antibody (IgG). Cell migration speed was then determined as for A. Data are represented as box and whisker plots (whiskers: 10-90 percentile), *n*=3 independent experiments; ****P<0.0001; ns, not significant; Mann–Whitney test. (C) Conditioned medium was collected from iMEFs stably transfected with a vector encoding tRNA_i_^Met^ or an empty vector control. Conditioned medium was then incubated with iMEFs and the migration speed of the cells determined as for A. Data are represented as box and whisker plots (whiskers: 10-90 percentile), *n*=3 independent experiments; ns, not significant; Mann–Whitney test. (D) iMEFs expressing tRNA_i_^Met^ were transfected with siRNAs targeting collagen II (siRNA-Col2) or a non-targeting control (siRNA-nt), or iMEFs that had the collagen II gene disrupted using CRISPR (CRISPR-Col2) and their appropriate CRISPR control (CRISPR-Con) [see Clarke et al. (2016)] and their migration speed was determined as for A. Data are represented as box and whisker plots (whiskers: 10-90 percentile), *n*=3 independent experiments; ns, not significant; Mann–Whitney test.

An alteration to the tRNA repertoire that is associated with highly aggressive cancer cells is elevated levels of the initiator methionine tRNA, tRNA_i_^Met^. As we have previously shown that elevated tRNA_i_^Met^ levels do not lead to upregulated protein synthesis, increased cell proliferation (Clarke et al., 2016), or altered energy metabolism (data not shown) we looked at the ability of this tRNA to influence other cell characteristics that are associated with cancer aggressiveness. Initially we compared the migratory behaviour of immortalised mouse embryonic fibroblasts (iMEFs), which overexpress tRNA_i_^Met^ (iMEF.tRNA_i_^Met^), with those expressing an empty vector as control (iMEF.Vector). Two pairs of iMEF pools were used throughout the subsequent experiments, with pools 1 and 2 having approximately 15- and 5-fold increases in tRNA_i_^Met^ expression, respectively (Clarke et al., 2016), which corresponded to the range of increased tRNA expression seen in human tumours compared to normal tissue (Pavon-Eternod et al., 2009). Overexpression of tRNA_i_^Met^ significantly increased speed of fibroblast migration both into scratch-wounds, and when subconfluent cells were moving randomly on plastic surfaces (Fig. 1A). We used siRNA and function-blocking antibodies to investigate which adhesion receptors were responsible for this altered cell motility. We deployed the mAb16 and 16G3 inhibitory monoclonal antibodies which recognise the receptor/ligand binding sites on α5 integrin and fibronectin, respectively, or we used siRNA to suppress levels of α5 integrin itself (Fig. S1A). Blockade of α5β1-fibronectin interaction with mAb16 or 16G3, or siRNA knockdown of α5 integrin (using either a SMARTPool or two individual siRNA oligos) suppressed migration of tRNA_i_^Met^ overexpressing fibroblasts, without affecting motility of the appropriate control vector-expressing cells (Fig. 1B).

We have previously shown that increased levels of tRNA_i_^Met^ leads to increased synthesis and secretion of ECM proteins, in particular type II collagen, and that this is responsible for tumour angiogenesis and growth (Clarke et al., 2016). To determine whether tRNA_i_^Met^ drives cell migration via synthesis of secreted and/or ECM factor(s), we collected conditioned medium from iMEF.tRNA_i_^Met^ and iMEF.Vector cells and tested the ability of this to influence fibroblast migration. Moreover, we suppressed type II collagen using siRNA or CRISPR (Clarke et al., 2016) and determined whether this influenced tRNA_i_^Met^-driven cell migration. However, neither conditioned medium from tRNA_i_^Met^-overexpressing cells (Fig. 1C), nor siRNA or CRISPR of type II collagen (Fig. 1D), altered the ability of tRNA_i_^Met^ to promote cell migration. Taken together, these data indicate that tRNA_i_^Met^ drives cell migration via mechanisms that are dependent on engagement of α5β1 integrin, but independent from the role that this tRNA plays in controlling the ECM synthesis and secretion.

**Fig. 2.**
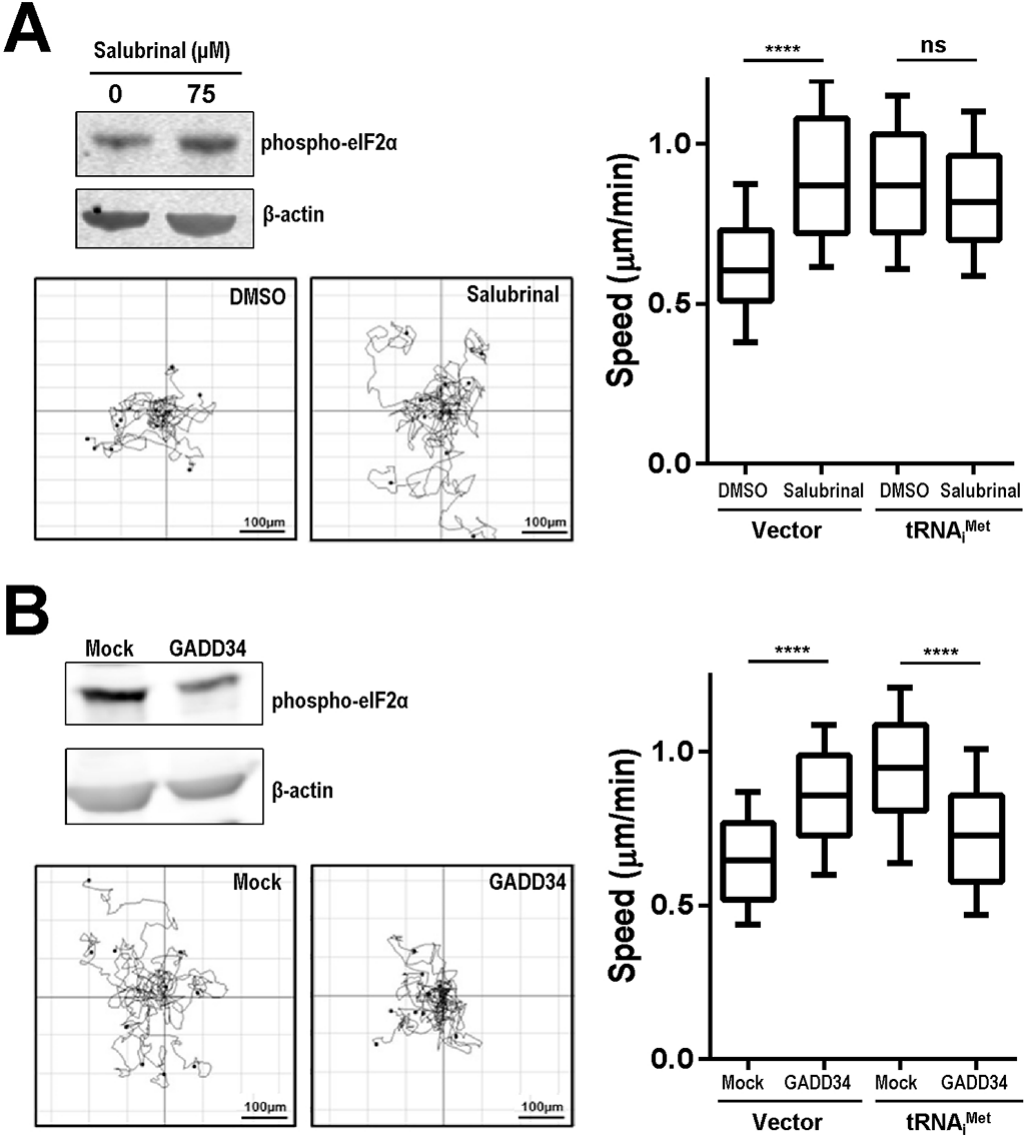
**tRNA_i_^Met^-driven cell migration is dependent on translation initiation ternary complex formation.** iMEFs stably expressing tRNA_i_^Met^ or vector control (Vector) were treated with salubrinal (75 μM) or vehicle control (DMSO) for 2 h (A). Alternatively these cells were transfected with a vector encoding the phosphatase, GADD34 or mock control (B). iMEF migration speed was then determined as for Fig. 1A. The trackplots indicate representative migration trajectories of these cells over a 17 h period. The western blots indicate the influence of salubrinal and GADD34 overexpression on levels of phosphorylated eIF2α. Data are represented as box and whisker plots (whiskers: 10-90 percentile); *n*=3 independent experiments; ****P<0.0001; ns, not significant; Mann–Whitney test. Scale bar: 100 μm.

The role of tRNA_i_^Met^ in translation initiation is dependent on its ability to associate with initiation factor eIF2 and GTP to form the ternary complex (Sonenberg and Hinnebusch, 2009), and translation initiation can be controlled by phosphorylation of eIF2α. Although we found there to be no consistent difference in phospho-eIF2α levels following tRNA_i_^Met^ overexpression (data not shown), indicating that cell migration was not dependent on absolute differences in phospho-eIF2α levels, we still considered it possible that tRNA_i_^Met^ levels may influence cell migration by altering ternary complex availability. To investigate the dependency of tRNA_i_^Met^-driven cell migration on TC levels, we used the phosphatase inhibitor, salubrinal, to increase levels of phospho-eIF2α by inhibiting phospho-eIF2α dephosphorylation (Fig. 2A) (Boyce et al., 2005). Conversely, we overexpressed the protein phosphatase regulatory subunit, GADD34, to decrease levels of phospho-eIF2α by recruiting protein phosphatase 1 to dephosphorylate phospho-eIF2α (Fig. 2B) (Boyce et al., 2005). Salubrinal treatment increased migration speed of iMEF.Vector cells, but not tRNA_i_^Met^ overexpressing cells (Fig. 2A), whereas expression of GADD34 completely reversed the ability of tRNA_i_^Met^ to promote cell migration (Fig. 2B). Taken together, these data indicate that tRNA_i_^Met^ drives cell migration in a way that is dependent on levels of the translation initiation ternary complex.

### Increased levels of tRNA_i_^Met^ drive cell migration *in vivo*

**Fig. 3.**
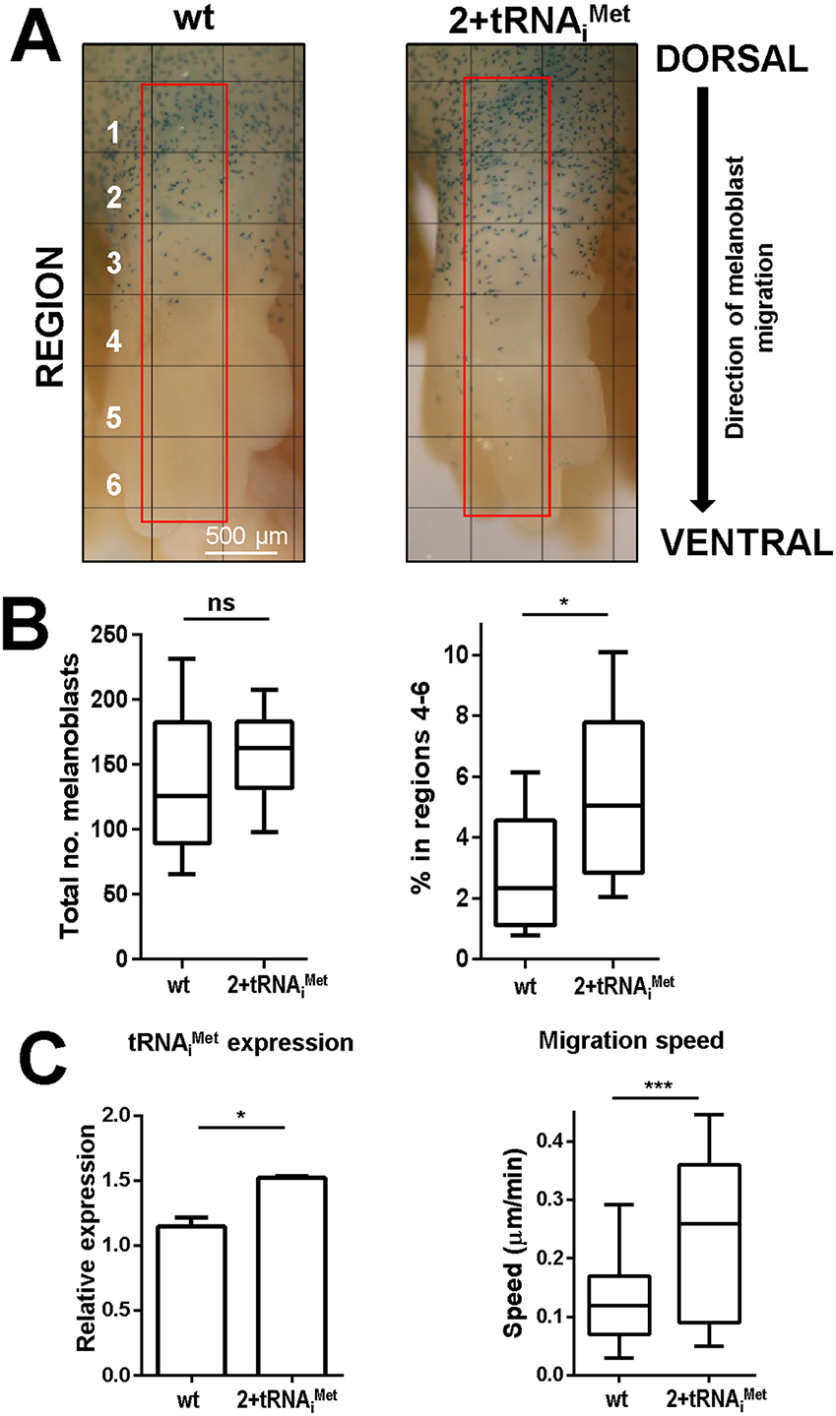
**Elevated expression of tRNA_i_^Met^ drives melanoblast migration in the developing embryo.** (A) Wild-type (wt) and 2+tRNA_i_^Met^ mice were crossed with a line expressing β-galactosidase under a melanoblast-specific promoter (DCT-LacZ). Embryos were removed at E13.5 and stained for β-galactosidase expression to visualise melanoblasts (A), and the total number (B; left panel) and proportion of β-galactosidase-positive cells within regions 4–6 (B; right panel) of the forelimbs was scored (B, left and right panels). Data are represented as box and whisker plots (whiskers: 10-90 percentile); *P<0.05; ns, not significant; Mann–Whitney test. (C) Wild-type and 2+tRNA_i_^Met^ mice were crossed with animals that were null for p16INK4A and that expressed a mutant allele of NRas under the melanoblast-specific tyrosinase promoter (Tyr-Nras^Q61K^; INK4a^−/−^). Melanocyte cell lines derived from the early pup skin of these Tyr::Nras^Q61K/°^; INK4a^−/−^; wild-type (wt) and Tyr::Nras^Q61K/°^; INK4a^−/−^; 2+tRNA_i_^Met^ mice were plated onto plastic surfaces and their migration determined using time-lapse microscopy as for Fig. 1A. Data are represented as box and whisker plots (whiskers: 10-90 percentile) (right panel) and as mean±s.e.m. (left panel) as indicated; *P<0.05; ***P<0.001; Mann–Whitney test.

Having used fibroblast models to establish a role for tRNA_i_^Met^ in cell motility, we wished to determine whether such a relationship could be demonstrated *in vivo* using a transgenic mouse possessing two additional copies of the tRNA_i_^Met^ gene (2+tRNA_i_^Met^ mouse) and which expresses increased tRNA_i_^Met^ levels in all tissues tested (Clarke et al., 2016). We looked at the distribution of melanoblasts (neural crest-derived precursors of melanocytes) which migrate dorso-ventrally during embryogenesis to populate the skin of the mouse (Thomas and Erickson, 2008). This process occurs between embryonic days (E)10–15 of embryogenesis, and by E13.5 the melanoblasts are *en route* to the limb extremities and other more ventral regions of the embryo. We crossed 2+tRNA_i_^Met^ mice to a line expressing DCT-LacZ (to visualise melanoblasts), and determined the proportion of cells that had moved to the distal/ventral regions of the forelimbs at E13.5 (Fig. 3A). Whilst there was no significant difference in the total melanocyte number – indicating no effect of tRNA_i_^Met^ overexpression on cell proliferation and/or differentiation – a higher proportion of melanoblasts had migrated to the limb extremities (regions 4-5 in Fig. 3A) of 2+tRNA_i_^Met^ mice than in their littermate controls (Fig. 3B). Given our previous observations that the contribution made by increased tRNA_i_^Met^ levels to tumour growth is not cell autonomous, it was necessary to determine whether the influence of tRNA_i_^Met^ overexpression on melanoblast migration was owing to the migratory behaviour of the cells themselves. To test this, we isolated melanoblasts from 2+tRNA_i_^Met^ embryos and measured their migration *ex vivo*. To enable growth of mouse melanoblasts in *ex vivo* culture we used mice that were null for INK4a and that expressed an allele of mutant NRas under a melanoblast-specific promoter (Tyr::Nras^Q61K/°^; INK4a^−/−^) (Lindsay et al., 2011). Melanoblasts isolated from 2+tRNA_i_^Met^ mice had elevated levels of tRNA_i_^Met^ and migrated faster than cells from their littermate controls, indicating that the ability of tRNA_i_^Met^ to promote *in vivo* migration of melanoblasts is likely to be cell-autonomous (Fig. 3C). Taken together, these data indicate that although increased levels of tRNA_i_^Met^ do not affect cell growth proliferation or differentiation, this transfer RNA plays a key role in controlling melanoblast migration velocity *in vivo* and *ex vivo*.

### Increased levels of tRNA_i_^Met^ drive melanoma invasion and lung colonisation

**Fig. 4.**
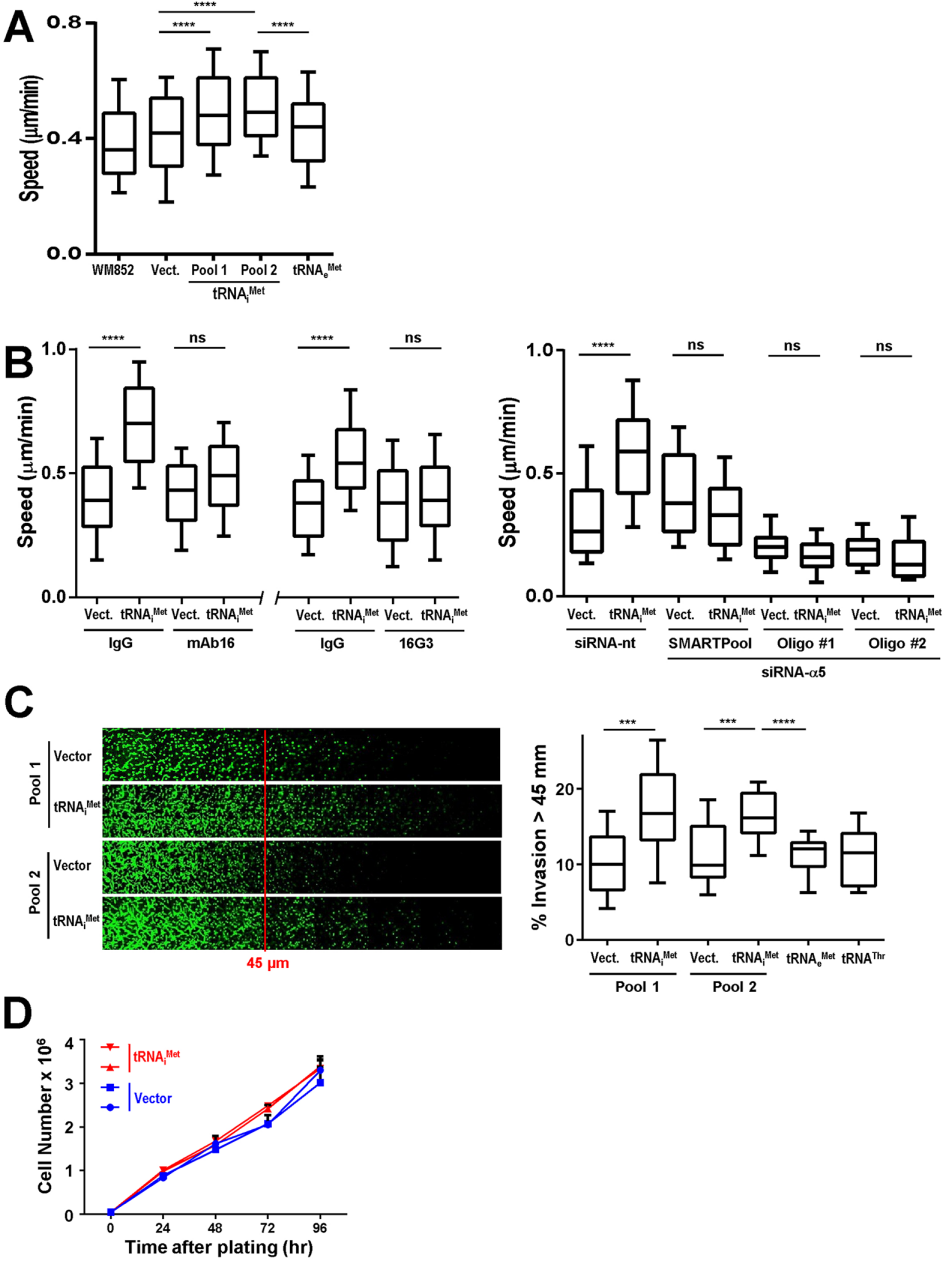
**Elevated levels of tRNA_i_^Met^ promote migration and invasion of melanoma cells.** (A) Untransfected WM852 melanoma cells (WM852) or those stably transfected with a vector encoding tRNA_i_^Met^ (two independent pools), tRNA_e_^Met^, or an empty vector control (Vect.) were plated onto plastic dishes and their migration speed determined as for Fig. 1A. (B) Control (Vect.) and tRNA_i_^Met^-expressing WM852 cells (pool 2 from A) were transfected with siRNAs targeting α5 integrin (siRNA-α5; either a SMARTPool or two individual siRNA oligonucleotides as indicated) or a non-targeting control (siRNA-nt) (right panel), or were left untransfected (left and centre panels). Migration of these cells was then determined as for Fig. 1A in the presence and absence of an α5β1 integrin blocking antibody (mAb16), an antibody which blocks integrin-fibronectin association (16G3) or the appropriate isotype-matched control antibodies (IgG). (C) WM266.4 melanoma cells were stably transfected with a vector encoding tRNA_i_^Met^ (two independent pools), tRNA_e_^Met^, tRNA^Thr^ or empty vector (Vect.) (two independent pools). Cells were allowed to migrate into Matrigel plugs towards a gradient of EGF and serum for 72 h, and then visualised by Calcein-AM followed by confocal microscopy. Optical sections were taken every 15 μm and consecutive images are displayed as a series running from left to right (C; left panels). Cell invasion beyond 45 μm was quantified (C; right panel). Data in A-C are represented as box and whisker plots (whiskers: 10-90 percentile); ****P<0.0001; ***P<0.001; ns, not significant; Mann–Whitney test. All data are from at least 3 independent experiments with multiple internal replicates. (D) WM266.4 cells stably transfecting with a control vector (Vect.) or tRNA_i_^Met^ (two independent pools of each) were plated onto plastic dishes and their rate of proliferation over a 96 h period was determined by cell counting. Values are mean±s.e.m., *n*=3.

A recent study indicates that there are key similarities between the molecular machinery involved in the migration of melanoblasts and the drive to metastasis in melanoma (Lindsay et al., 2011), prompting us to investigate the role of tRNA_i_^Met^ in the migratory and invasive behaviour of melanoma. Initially we looked at the WM852 melanoma cell line and found that the migration speed of these cells was significantly enhanced by overexpression of tRNA_i_^Met^ (in two independent stable pools of cells), but not by the elongator methionine tRNA, tRNA_e_^Met^ (Fig. 4A; Fig. S2A). Moreover, as for fibroblast migration, the ability of tRNA_i_^Met^ to influence melanoma cell movement was completely dependent on α5β1 integrin (as determined using blocking antibodies and siRNA with a SMARTPool and two individual siRNA oliogonucleotides) and its engagement with fibronectin (Fig. 4B; Fig. S1B). To look at the invasive and metastatic characteristics of melanoma cells we used the WM266.4 cell line, which was established from a cutaneous melanoma metastasis and exhibits moderately invasive behaviour both *in vivo* and *in vitro*. WM266.4 cells invade into Matrigel and elevated expression of tRNA_i_^Met^ increased invasiveness in two independent pools of WM266.4 cells, whereas other Pol III products (tRNA_e_^Met^ and a tRNA for threonine, tRNA^Thr^) were ineffective in this regard (Fig. 4C; Fig. S2B). Furthermore, overexpression of tRNA_i_^Met^ did not influence proliferation of WM266.4 cells, indicating that tRNA_i_^Met^'s ability to drive melanoma cell invasion is not mediated via increased cell growth (Fig. 4D).

**Fig. 5.**
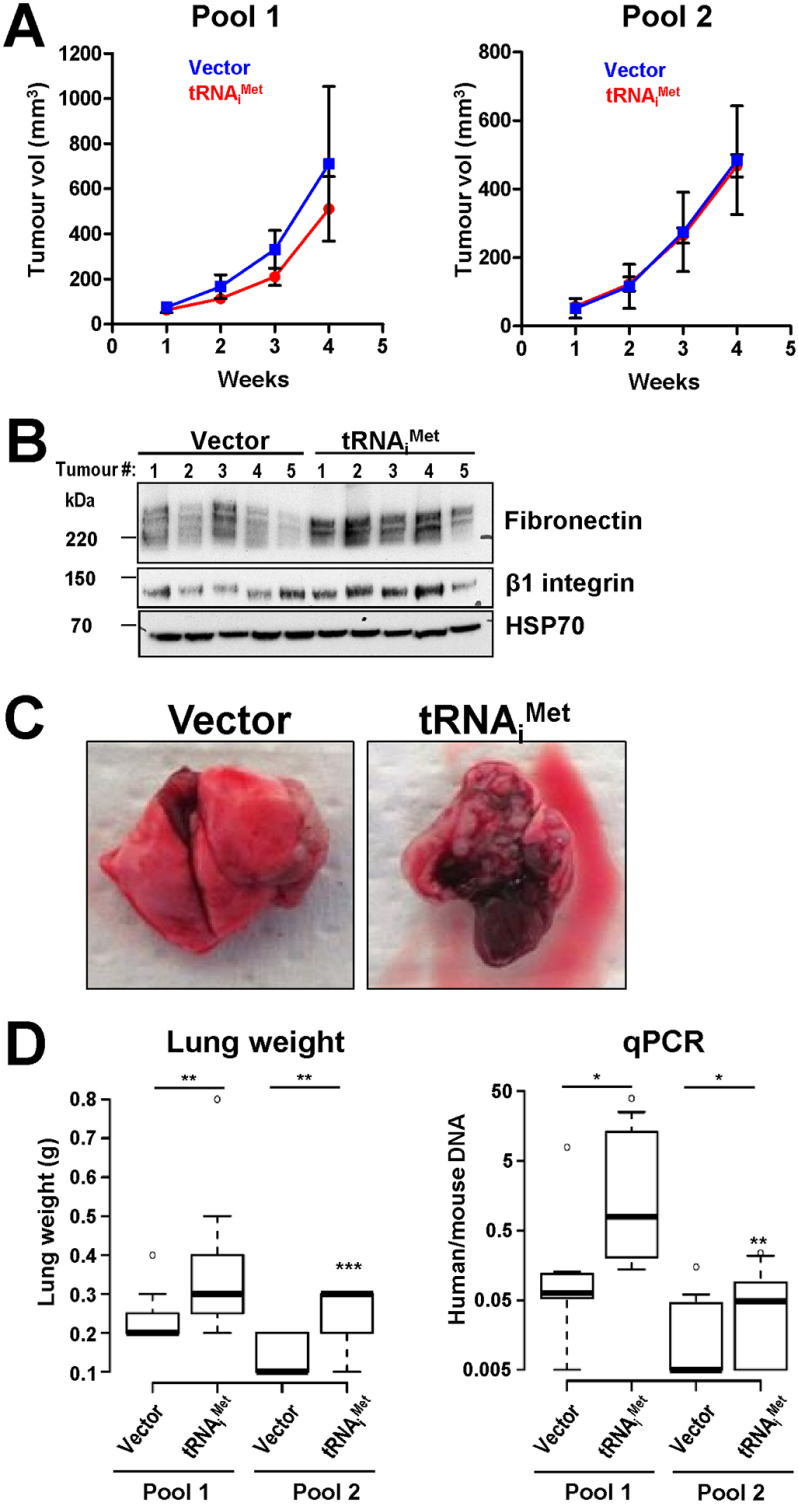
**tRNA_i_^Met^ drives melanoma metastasis, but not primary tumour growth.** (A) WM266.4 cells stably expressing a vector encoding tRNA_i_^Met^ (two independent pools) or an empty vector (Vector) (two independent pools) and were injected subcutaneously into the flank of CD1 nude mice. Subcutaneous tumour growth was measured by callipers three times a week and tumour volume was calculated from these. Values are mean±s.e.m. (B) tRNA_i_^Met^ and empty vector expressing WM266.4 cells were injected subcutaneously into CD1 nude mice. The resulting tumours (5 from each condition) were lysed and their fibronectin and β1 integrin content determined by western blotting. HSP70 is used as a loading control. (C,D) tRNA_i_^Met^ and empty vector expressing WM266.4 cells (two independent pools of each as for A) were injected via the tail vein into CD1 nude mice. The lungs of these animals were assessed for the presence of tumours by visual inspection (C), by determination of lung weight (*n*=7+7 pool 1 and 7+9 pool 2, pLHCX and tRNA_i_^Met^ respectively) (D, left panel), and by qPCR to quantify the proportion of human genomic DNA (from the WM266.4 cells) with respect to mouse genomic DNA (from the host animal) (D, right panel). *n*=7+7 pool 1 and 7+11 pool 2, vector and tRNA_i_^Met^ respectively. Values are expressed as box and whisker plots (whiskers: 5-95 percentile). The ratios of human to mouse DNA are expressed on a Log^10^ scale. **P<0.01; *P*<0.05; van Elteren Test (stratified Mann–Whitney). N.B. The injection of cells from pool 1 and pool 2 were conducted in experiments that were independent from one another.

We then studied the influence of tRNA_i_^Met^ on tumour growth and lung colonisation using subcutaneous and intra-tail vein xenograft approaches, respectively. Overexpression of tRNA_i_^Met^ did not influence growth of tumours formed following subcutaneous injection of WM266.4 cells, consistent with a lack of a regulatory role for this Pol III product in cell growth and proliferation (Fig. 5A). However, western blotting indicated that there was a tendency for the subcutaneous tumours formed from tRNA_i_^Met^-overexpressing cells to have elevated fibronectin and β1 integrin content, suggesting that tRNA_i_^Met^ may drive α5β1 integrin-dependent processes in these tumours (Fig. 5B). As tumour cell invasiveness can be promoted by α5β1 and is often associated with increased fibronectin levels, we injected WM266.4 cells overexpressing tRNA_i_^Met^ or a control vector (two independent pools of each) into CD1 nude mice via the tail vein and measured the capacity of these cells to colonise the lung. Upon visual inspection, the lungs of mice injected with tRNA_i_^Met^-overexpressing WM266.4 cells contained more tumours and were heavier than those from animals injected with control cells (Fig. 5C,D). To quantify lung-tumour burden we used qPCR to determine the ratio of human (from the WM266.4 cells) to mouse (the host) DNA. This indicated that the tumour burden in the lungs from the animals injected with tRNA_i_^Met^-overexpressing cells was two- to threefold higher than their appropriate controls (Fig. 5D). Taken together, these data indicate that increased levels of the Pol III product, tRNA_i_^Met^ is sufficient to promote the ability of melanoma to invade into and to colonise tissues without influencing the proliferation and growth of the primary tumour.

**Fig. 6.**
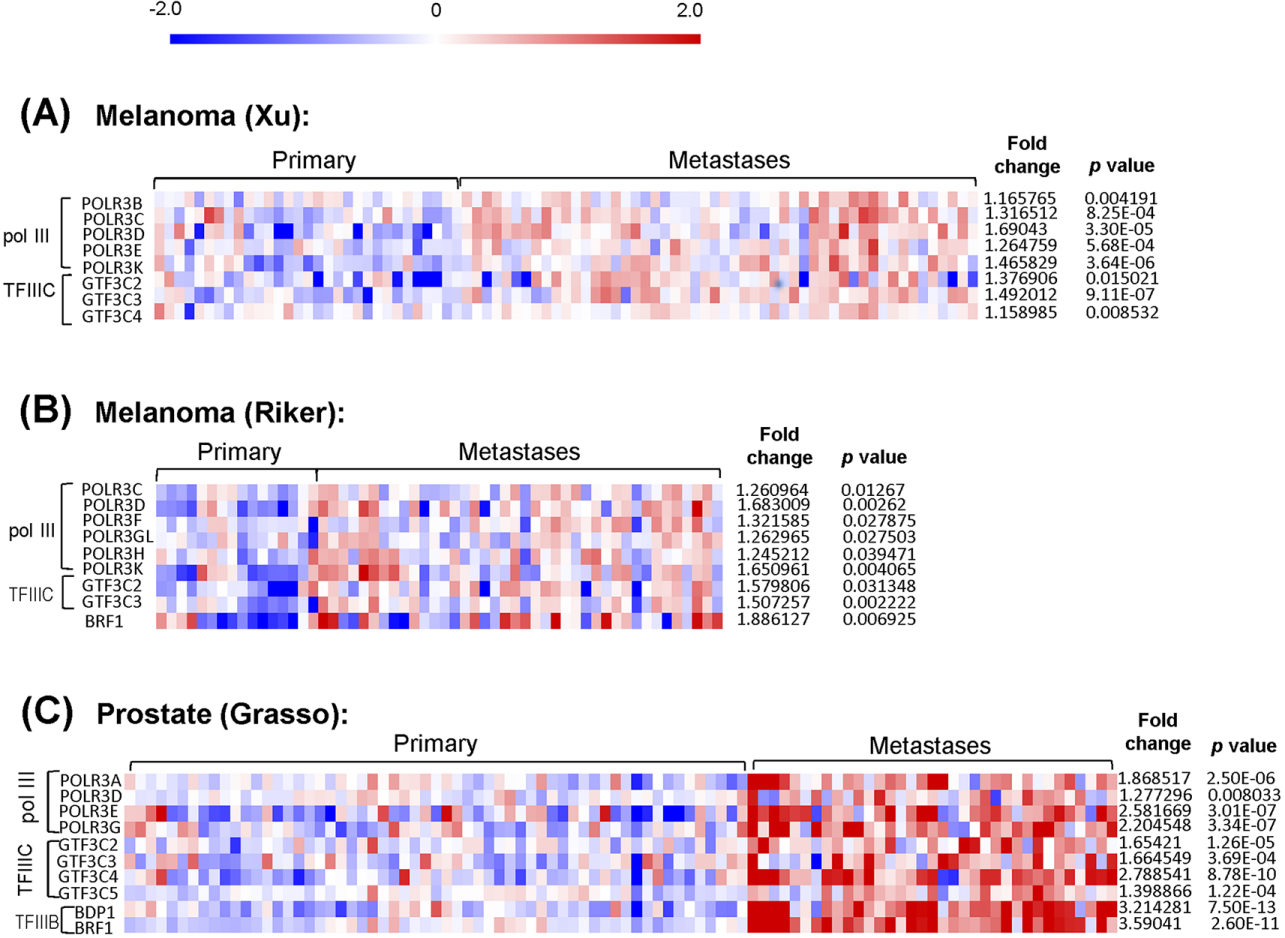
**Pol III and its products are associated with invasion and metastasis.** Heat maps from Oncomine data sets showing pol III (POLR3), TFIIIC (GTF3C) and TFIIIB (Brf1, BDP1) subunit mRNA expression in primary tumours versus patient-matched metastases in melanoma (Xu and Riker datasets; A,B) or prostate cancer (Grasso dataset; C). The heatmap scale bar is indicated at the top, and the average degree of upregulation of transcripts in metastasis and statistical significance values of these are tabulated on the right-hand side. In Oncomine, all data are normalised as follows: negative values were not included and all data were log^2^-transformed, median-centred per array, and the standard deviation was normalised to one per array. Depending on the type of microarray (one or two colours) data are presented either as log^2^ median-centered intensities (Xu and Riker), or log^2^ median-centered ratios (Grasso). The scale bar is log^2^.

We wished to determine whether tRNA_i_^Met^ and other related Pol III products were associated with invasion and metastasis in human cancer. However, because we have been unable to obtain comprehensive bioinformatic data concerning the levels of tRNA_i_^Met^ in large cohorts of human tumours, we analysed data from a number of publically available datasets to look at the levels of other transcripts associated with Pol III in melanoma and prostate cancer metastases, and compared these with the corresponding levels in patient-matched primary tumours. mRNAs encoding for Pol III subunits and for a Pol III's transcription factor, TFIIIC were increased by 1.3 to 3.5-fold in metastases from melanoma by comparison with patient-matched primary tumours in at least two (Xu and Riker) independent datasets (Fig. 6A,B). Moreover, similar upregulation of Pol III-related transcripts in prostate metastases was observed in the Grasso dataset (Fig. 6C). Taken together, these data indicate that a number of Pol III products are upregulated as tumours metastasise and that one product in particular, tRNA_i_^Met^, is responsible for driving the cell migratory and invasive characteristics that leads to tumour dissemination and metastasis.

## DISCUSSION

In this paper we show that elevated levels of tRNA_i_^Met^ activate a cell migration programme dependent on α5β1 integrin and formation of the translation initiation ternary complex. Furthermore, we show that migration and invasion driven by this alteration to the tRNA repertoire may be demonstrated *in vivo*, and leads to upregulation of embryonic melanoblast migration and increased metastasis in a xenograft model of melanoma. Importantly, tRNA_i_^Met^-dependent invasiveness is achieved without any detectable alteration to primary tumour growth or cell proliferation, indicating that the tumour cell migratory machinery is likely to be a key target of the tRNA repertoire during the metastatic cascade.

The activity of Pol III has been known for some time to be associated with cancer and Pol III transcriptional programmes are regulated by a number of oncogenes and tumour suppressors (White, 2008). However, although all tRNAs are transcribed by Pol III there is considerable selectivity in their expression leading to association of particular tRNA repertoires with cells that are either actively proliferating or those that are more differentiated and/or quiescent (Gingold et al., 2014). A combination of increased Pol III activity and epigenetic modification of tRNA genes leads to a proliferative tRNA repertoire that is characterised by particularly high levels of tRNA_i_^Met^. Moreover, tRNA_i_^Met^ levels are upregulated in stromal cells such as carcinoma-associated fibroblasts (Clarke et al., 2016). This had initially been proposed to reflect a need for global upregulation of translation initiation to support increased protein synthesis in rapidly growing cells; however, our data suggest that this is not the case, and that selective upregulation of tRNA_i_^Met^ contributes to cancer via two distinct mechanisms, neither of which directly involve altered cell proliferation or growth, but which differ depending on the whether the tRNA is elevated in the stromal or cancer cell compartments. Thus, if tRNA_i_^Met^ levels are elevated in stromal fibroblasts, this leads to highly significant and selective alterations to their secretome which drive tumour growth by increasing endothelial cell motility to promote tumour angiogenesis (Clarke et al., 2016). More specifically, this is attributable to selective upregulation of synthesis and secretion of type II collagen. By contrast, upregulation of tRNA_i_^Met^ in tumour cells leads to acquisition of migratory and invasive characteristics that are cell autonomous and independent from components of the tRNA_i_^Met^-driven secretome.

One possible mechanism for tRNA_i_^Met^'s cell-autonomous drive to cell migration is via generation of microRNA-like molecules. Recent reports indicate the existence of a class of tRNA-derived small RNAs that interact with the RNAi machinery, possibly to influence mRNA stability and translation (Pederson, 2010). However, microRNAs are normally thought to suppress cell migration and other characteristics imputed to cancer cells, as a microRNA-like molecule derived from the 3′ end of a mature tRNA is suppressed in B cell lymphoma (Maute et al., 2013). Moreover, our data indicate that tRNA_i_^Met^ controls cell migration via a mechanism involving its canonical role in translation initiation. Indeed, we show that levels of the translation initiation ternary complex (of which tRNA_i_^Met^ is a component) are key to control of cell migration. Elevated levels of the ternary complex leads to increased migration speed, and overexpression of GADD34 (to reduce ternary complex levels) completely opposes tRNA_i_^Met^-driven cell migration without affecting movement of cells that do not have elevated levels of tRNA_i_^Met^.

tRNA_i_^Met^'s influence on stromal ECM deposition is associated with upregulated synthesis of a defined cohort of proteins (Clarke et al., 2016). Therefore, we considered whether tRNA_i_^Met^'s ability to drive cell migration may involve altered synthesis of particular proteins. Indeed, although the majority of tRNA_i_^Met^'s translational targets are secretory and/or ECM proteins (and we don't find tRNA_i_^Met^'s drive to cell migration to rely on any of these), there are a few cellular proteins whose synthesis is promoted by increased levels of tRNA_i_^Met^ (Clarke et al., 2016). Despite these efforts, we have been unable to identify any individual component of the cellular tRNA_i_^Met^ translatome with a clear role in the ability of this tRNA to drive cell migration and invasion. Therefore, we feel that the connection between tRNA_i_^Met^ and the cell migration machinery might be mediated somewhat indirectly. Clearly, elevated levels of tRNA_i_^Met^ are associated with a net shift towards increased production of secreted proteins, and this would be expected to be associated with increased flux of membrane and protein traffic through elements of the biosynthetic secretory pathway, such as the endoplasmic reticulum (ER), Golgi and post-Golgi compartments. Such generally increased delivery of membrane and protein traffic to the plasma membrane would be likely to influence cell migration. Indeed, all of the receptors that drive cell migration and invasion are delivered to the cell surface via the trans-Golgi network, and more recently, integrins have been shown to recycle via a pathway involving elements of the ER and the trans-Golgi network (Reverter et al., 2014; Shafaq-Zadah et al., 2016). Further work will be necessary to determine the consequences of upregulated biosynthetic secretory traffic on the trafficking of pro-invasive adhesion receptors, and whether this contributes to tRNA_i_^Met^'s cell-autonomous drive to cancer cell invasiveness.

Many of the components of the tRNA_i_^Met^-driven secretome are large proteins which, when synthesised in large quantities, would be expected to put considerable demands on the biosynthetic and secretory machinery. Thus it is likely that the protein-folding and post-translational capacity of the ER is stretched by the considerably increased production of collagens which is enlisted following tRNA_i_^Met^ upregulation, and if so, this would elicit ER stress responses and other cell signalling pathways known to relay information from the ER to the nucleus. Indeed, ER stress is well-established to influence transcriptional programmes that lead to metastasis (Bu and Diehl, 2016), and it will be interesting to determine whether this is in part responsible for connecting increased tRNA_i_^Met^'s levels to the metastatic capacity of cancer cells.

To conclude, we have identified two distinct, but complementary, mechanisms through which a defined alteration to the tRNA repertoire may drive tumour growth and metastasis. Upregulation of tRNA_i_^Met^ in stromal fibroblasts drives synthesis of a collagen II rich ECM which supports angiogenesis and primary tumour growth (Clarke et al., 2016), whilst elevated levels of tRNA_i_^Met^ in the tumour cells influences their migratory phenotype to increase their propensity to metastasise. These studies combine to provide a body of experimental evidence which link alterations to the tRNA repertoire to tumour progression *in vivo*, and further investigation into these processes will likely contribute to effective strategies to oppose cancer progression and metastasis in humans.

## MATERIALS AND METHODS

### Cell culture

WM266.4, WM852 and iMEF lines were cultured in Dulbecco's Modified Eagle Medium (Life Technologies) supplemented with L-glutamine (200 µM) and 10% foetal calf serum. Stable pools were created by transfection with a pLHCX vector which had had its pCMV promoter removed via restriction digest to allow correct expression of the inserted tRNAs from their internal promoters. Pools were selected for and subsequently in maintained in 250 µg/ml Hygromycin.

Melanocyte culture was conducted at 2 days postnatal. Pups were placed in ice cold 70% ethanol for no longer than 30 s. Skin was dissected off, cut into pieces and incubated with 1.5 ml each of collagenase type 1 and 2 at 37°C, 5% CO_2_ for 40 min in 6-well plates. Contents were transferred in to 15 ml wash buffer (1× HBSS, 1 mM CaCl_2_, 0.005% DNase1) and centrifuged at 218 ***g*** (1100 rpm) for 5 min. Pellets were resuspended in dissociation buffer and incubated at 37°C, 5% CO_2_ for 10 min. Tissue was then passed through an 18 G needle and transferred to 10 ml wash buffer and allowed to settle. Grease and fur was removed from top of buffer and samples spun at 218 ***g*** (1100 rpm) for 5 min. Cells were washed in PBS and then plated in 6-well plates at 1×10^6^ per well in Hams F-12 media (Life Technologies) with 10% FCS and 200 nM TPA. To selectively reduce fibroblast growth, 50 µg/ml G418 was added to the media after 2 days.

For cell proliferation assays 10,000 cells/well were plated in 6-well tissue culture plates (Falcon, Corning Inc) and total cell number counted 24, 48, 72 and 96 h post plating (Casy Cell Counter, Innovatis AG). In each assay, the media was refreshed every 48 h and each well counted three times, with the average value being recorded, experiment repeated three times.

### Generation of the 2+tRNA_i_^Met^ mice

Two copies of murine tRNAi^Met^ gene (trna78), including ∼140 bp flanking sequences were targeted into the HPRT locus of HM1 embryonic stems cells which were used to generate chimeric mice from which tRNAi^Met^ trangenic offspring were derived by crossing to C57Bl/6 mice. The generation of these mice and the breeding strategy for this is described in Clarke et al., (2016).

### Generation of tRNA expression vectors

To produce vectors without an exogenous pol II promoter, fragments containing tRNA_i_^Met^, tRNA_e_^Met^, and tRNA^Thr^ were cloned into the multiple cloning site of pLPCX using EcoR1. In parallel, pLHCX was digested with HpaI and SnaBI to remove the pCMV promoter. The tRNA containing pLHPX vectors were then digested with BglII and ClaI to release the tRNA containing fragments and these were subsequently cloned into the promoterless pLHCX vector that had been digested with BglII and ClaI.

### Metabolic labelling

Cells were grown to 80-90% confluence, washed 2× in PBS and then 5 ml DMEM (methionine and cysteine) containing 10% dialysed foetal calf serum and L-glutamine to deplete cells of methionine and cysteine. Cells were incubated for 30 min, 37°C, 5% CO_2_. The medium was aspirated and replaced with 2 ml of pulse labelling solution (as medium above but with 0.22mCi EasyTag added) and cells incubated for 20 min, 37°C, 5% CO_2_, washed with cold PBS and then scrapped into ice-cold PBS into 15 ml falcons. 20 µl of cell suspension was added to BSA/NaN_3_ (1 mg/ml BSA in 0.02% sodium azide) and TCA precipitated in 1 ml ice cold TCA, vortexed and incubated 30 min on ice. 20 µl of cell suspension was also kept for total count and the rest of the cells spun and lysed for measurement of total protein concentration. The precipitated material was filtered on to Whatman GF/C 2.5 cm filter paper, and the filters washed with 2×5 ml ice cold 10% TCA, 2×5 ml ethanol, and then air dried for 30 min. The cell suspension kept back for total count was spotted on to filter paper and dried. Scintillation fluid was added and filters counted using a Microbeta Trilux scintillation counter (Perkin Elmer).

### Cell motility assays

For confluent monolayer scratch assays, cells were grown to confluence in 6-well dishes, scratched with a sterile pipette tip, washed and 4 ml of fresh media added. For subconfluent migration, cells were plated at approximately 2×10^5^ in 6-well plates. siRNA transfection was conducted 72 h prior to timelapse. Migration was captured by timelapse capturing images every 15 min (10 min for subconfluent iMEFs). Migration velocity was calculated using single cell tracking via ImageJ analysis.

For inverted invasion assays, Matrigel (BD biosciences) was polymerised in Transwell permeable inserts (Corning) at 37°C, 5% CO_2_ for 1 h. Inserts were inverted and 2-5×10^4^ cells seeded onto the filter surface. Cells were allowed to adhere for 2-3 h at 37°C, 5% CO_2_. Inserts were washed 3× in serum free medium. After final wash, inserts were placed in serum-free medium and medium plus 10% foetal calf serum added above the Matrigel to act as a chemo-attractant. Invasion assays were prepared 24 h after siRNA or plasmid transfection. After 72 h invading cells were stained with Calcein-AM and visualised via confocal microscopy through optical sections obtained in the z-plane at 15 µm intervals. Quantification was conducted using ImageJ Area calculator plugin.

### siRNA and vector transfection

5 nM (20 nM for integrin α5) siRNA were transfected into 5×10^5^ cells using Hiperfect (Qiagen) and pol III oligos (si 1 CAAGUAUGGUGACAUCGU, Ambion pre-designed s21945:, si 2 UCUAACCGUGGUUUCUCAAUUGGGA, Invitrogen custom Stealth RNA), TFIIIC (CAGUGAACGGAGAACGAU, Ambion pre-designed s6327), human integrin α5 (Thermo Scientific ON-TARGET SMARTpool L-008003-00; #1 which is UCACUACGCUCUCAACUUC; #2 which is ACACGUUGCUGACUCCAUU), mouse integrin α5 (Thermo Scientific ON-TARGET SMARTpool L-060502-01-0005; #1 which is ON-TARGET J-060502-09; #2 which is J-060502-11) or non-targeting oligonucleotides in 6-well plates. 2.5 µg of plasmid were transfected in 6-well plates using polyfect (Qiagen) for melanoma cell lines and human fibroblasts or TransIT (Mirus) for iMEF lines.

### Embryo analysis and β-galactosidase staining

Embryos were dissected at E13.5 after detection of a vaginal plug at E0.5 and fixed in 0.25% gluteraldehyde at 4°C for 45 min on a rolling platform. Embryos were washed in PBS at 4°C on rolling platform for 15 min followed by 3×30 min washes in 2 mM MgCl_2_, 0.01% Na-deoxycholate, 0.02% NP-40 in PBS at room temperature. β-galactosidase substrate [1 M MgCl_2_, 0.02% NP-40, 0.01% Na-deoxycholate, 0.04% X-Gal, 5 mM K_3_Fe(CN)_6_, 5 mM K_4_Fe(CN)_6_] was added and embryos incubated in darkness on rota overnight followed by incubation in 4% paraformaldehyde for 2 h at 4°C.

### qPCR analysis

cDNA was prepared with Qiagen Quantitech Reverse Transcription kit from 5, 10 or 20 ng of RNA extracted from cells using Trizol. qPCR was conducted using BioRad CFX platform, at annealing temperature of 60°C. Primer pairs: tRNAiMet F AGAGTGGCGCAGCGGAAG, R GAGGATGGTTTCGATCCATC; tRNA_e_^Met^ F CCTCTTAGCGCAGCGGGC, R GCCCTCTCTGAGGCTCGAACTC; tRNA^Ile^ F GGCGGCCGGTTAGCTCAG, R CCCGTACGGGGATCGAAC; tRNA^Tyr^ F CCTTCGATAGCTCAGCTGGTA, R AGCGACCTAAGGATGTCCGC; tRNA^Leu^ F ATGGCCGAGTGGTCTAAGG, R ACCAGAAGACCCGAACACAG; 5S F CAGCACCCGGTATTCCCAGG, R GGCATACCACCCTGAACGC.

Genomic DNA was extracted from 12.5 mg mouse lung tissue using Qiagen DNaeasy kit. The proportion of human genomic DNA was analysed by performing qPCR on 7 ng of lung DNA using human specific primers (F 5′-CATCCCTGGACTGATTGTCA, R 5′-GGTTGGCCAGGTACATGTTT) or mouse specific primers (F 5′-CAGCGCTAGAGTGAGCGGATTA, R 5′-GAATTGATCAGTCTGCAGACAGCC).

### Xenograft experiments

1×10^6^ were either subcutaneously or intravenously injected via the tail vein into CD1 nude mice. Subcutaneous tumour growth was measured by callipers three times a week until they reached a size endpoint of 15 mm. Intravenously injected mice were taken at a designated timed endpoint. All animal work was carried out with ethical approval (University of Glasgow and Institute of Cancer Research) in dedicated barrier animal facilities proactive in environmental enrichment and in accordance with the revised Animals (Scientific Procedures) Act 1986 and the EU Directive 2010/63/EU.
